# Synchronous Hyper-Accelerated Follicular (SHAF) Growth During Controlled Ovarian Stimulation: An Unusual Case Posing an Unexpected Challenge

**DOI:** 10.7759/cureus.91763

**Published:** 2025-09-07

**Authors:** Soumya R Panda, Kaninika Panda, Gyanendra Pradhan, Pratyasha Panda

**Affiliations:** 1 Reproductive Medicine, Santaan Fertility Centre and Research Institute, Bhubaneswar, IND

**Keywords:** asynchronous follicular growth, controlled ovarian stimulation, duostim protocol, shaf, synchronous hyper-accelerated follicular growth

## Abstract

Abnormal follicular development during ovarian stimulation is a worrisome factor for the fertility specialist. Here, we report a unique case in which all the ovarian follicles developed in a synchronous but unusually accelerated pattern, posing an exceptional challenge. After starting the ovarian stimulation, we noticed follicles as large as 20 mm, 18 mm, and 17 mm on day 5 of stimulation. As most of the follicles grew to such an extent simultaneously, we named this pattern of follicular growth synchronous hyper-accelerated follicular growth. Finally, we revised our initial management protocol and adopted an alternative approach (the dual stimulation protocol) to achieve the desired result. In the second phase of stimulation, we were able to retrieve three follicles, which resulted in the formation of two embryos (4AA and 3BB). Ultimately, we successfully achieved a live birth after transferring these embryos. We are the first authors to report a case presenting with such an abnormal follicular growth pattern.

## Introduction

A complex endocrine network in the human body governs follicular growth and regression in the ovary. The approaches to controlled ovarian stimulation for in vitro fertilization (IVF) are a favorite research interest among many scientists, and the field is being continuously explored. Follicular growth is not always uniform and can sometimes take an abnormal pattern, such as slow growth, rapid growth, asynchronous growth, premature rupture, or premature luteinization [1]. Although controlled ovarian stimulation aims to induce accelerated follicular growth, it can sometimes lead to a hyper-acceleration of follicular growth and an exaggerated ovarian response, which can disrupt normal physiological balances. The risks associated with such a response are ovarian hyperstimulation syndrome (OHSS), suboptimal oocyte and embryo quality, cycle cancellation or poor outcomes, multiple pregnancies and related risks, etc. [2,3]. Development of asynchronous follicular growth is a common phenomenon during stimulation with gonadotropins. This leads to the development of follicles at different stages of maturation and is a worrisome factor for clinicians.

In some cases, follicle growth is slow, whereas in others, an abnormally large follicle develops before the primary cohort reaches the minimum standard size for trigger injection [1]. Such incidents often lead to cancellation of the cycle; in fact, this was almost the norm in the era when pituitary downregulation was not a part of stimulation protocols. Here we are reporting a case where all the follicles grew in a synchronous but unusually accelerated growth pattern, thus posing a unique challenge.

## Case presentation

A 39-year-old female presented to our outpatient department with secondary infertility for 13 years. She had a history of multiple cycles of ovulation induction with oral ovulogens and also a history of two cycles of IUI treatment, all of which had failed. Her serum anti-Müllerian hormone (AMH) was 1.8 ng/ml; the baseline serum luteinizing hormone (LH), follicle-stimulating hormone (FSH), and all other hormonal profiles were within normal limits. The day 2 baseline ultrasonography revealed an antral follicle count of five. So an IVF cycle was planned for her. From day 2 of her next menstrual cycle, controlled ovarian stimulation was initiated with recombinant FSH 150 IU (Gonal-F, Merck Ltd., Feltham, UK) and highly purified human menopausal gonadotropin 75 IU (Gynogen, J B Chemicals and Pharmaceuticals Ltd., Worli, Mumbai). She was advised to attend stimulation day 5 for follicular monitoring. On the fifth day of follicular tracking, there was one dominant follicle on the right ovary and two on the left ovary. To our surprise, these were extremely large. The measurements of these follicles are provided in Table 1.

**Table 1:** Measurements of follicles in the first cycle of stimulation AFC: antral follicle count

Stimulation day	Menstrual day	Right ovary	Left ovary
1	2	AFC 2	AFC 3
5	6	18 mm (1); 10 mm (1)	20 mm (1); 17 mm (1); 11 mm (1)

After noticing such a large size of follicles, one shot of GnRH antagonist (Cetrolix, Intas Pharmaceuticals Ltd., Gujarat, India) was given immediately, and the dose of recombinant FSH was reduced to 75 IU. Serum estradiol (E2) level and serum progesterone (P4) were found to be 276 pg/ml and 0.6 ng/ml, respectively. Keeping in mind a plan of dual stimulation (Duostim), a GnRH agonist trigger (Decapeptyl 0.2 mg triptorelin, Ferring, Switzerland) was administered on the same day. Oocyte retrieval was performed according to the usual protocol, and two oocytes were retrieved (one M-II and one M-I). Small follicles were left alone. After doing IVF, one oocyte was fertilized. The resulting embryo was cultured till the 3rd day and was frozen as a pre-compact morula. Following administration of one shot of GnRH antagonist consecutively for three days, follicular stimulation was again started. This time, the starting gonadotropin dose was 150 IU. The growth of follicles is shown in Table 2.

**Table 2:** Measurement of follicles during the second phase of stimulation AFC: antral follicle count

Stimulation day	Menstrual day	Right ovary	Left ovary
1	11	AFC 4	AFC 3
4	14	11 mm (1); 10 mm (1)	13 mm (1); 11 mm (1)
6	16	14 mm (1); 13 mm (1); 10 mm (1)	16 mm (1); 13 mm (1)
8	18	17 mm (1); 15 mm (1); 12 mm (1)	20 mm (1); 16 mm (1)
9	19	19 mm (1); 18 mm (1); 14 mm (1)	22 mm (1); 18 mm (1)

**Figure 1:**
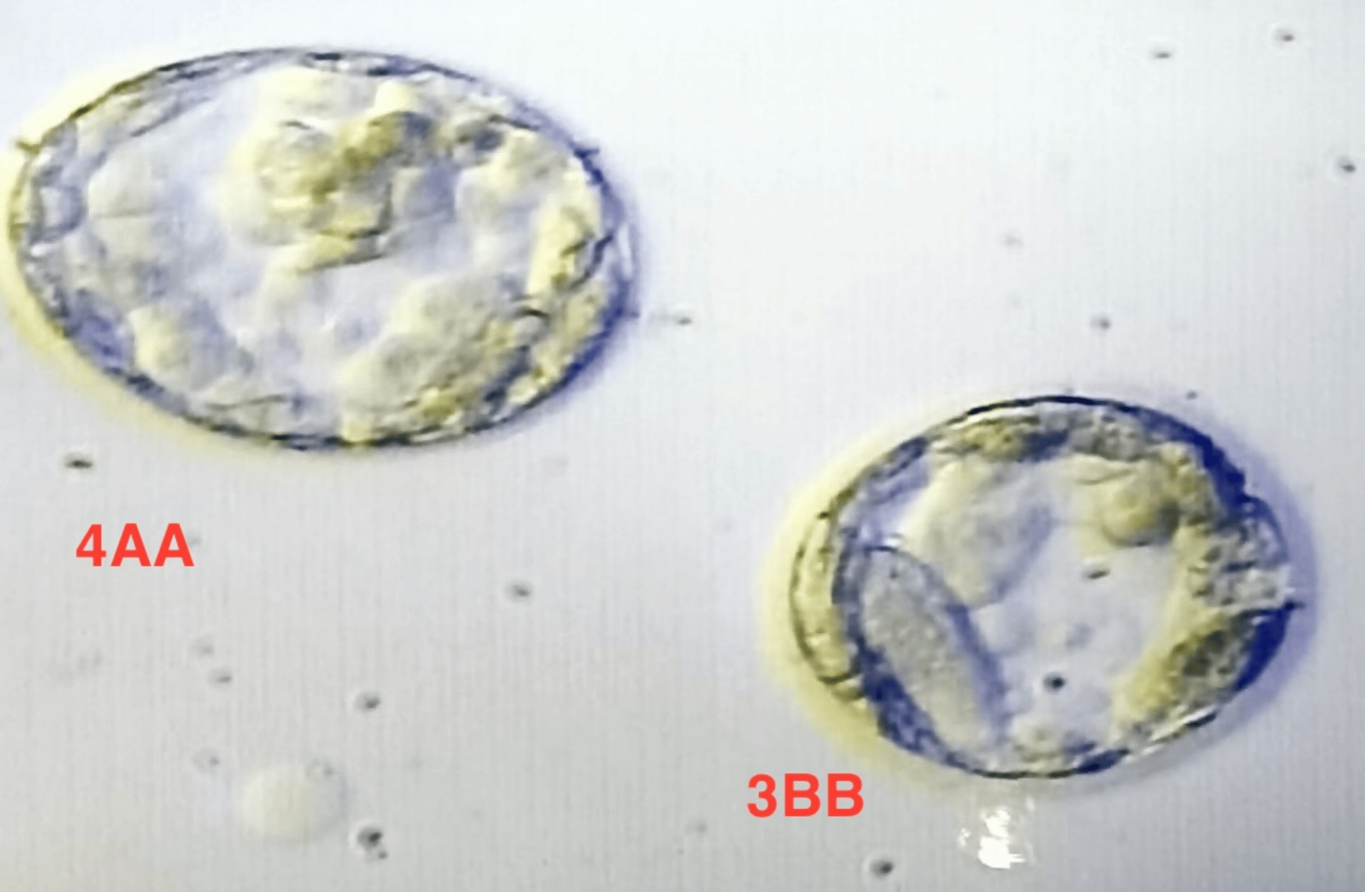
Image showing day 5 embryos (Grade 4 AA and 3BB) of the patient obtained from the second phase of stimulation 4AA and 3BB represent different grades of embryos.

Now, the trigger was given on the 9th day of stimulation, three oocytes (two M-II oocytes and one M-I oocyte) were retrieved, and after IVF, two oocytes were fertilized. The embryos were cultured till the fifth day, and the embryos (4AA and 3BB) were frozen (Figure 1).

Two months later, embryo transfer was planned, and two blastocysts (4AA and 3BB) were transferred, which resulted in a singleton pregnancy, and a healthy male child was delivered at the gestational age of 37 weeks and two days by cesarean section.

## Discussion

Overall, the rate of growth of ovarian follicles is 1.4 mm/day in spontaneous menstrual cycles and 1.7 mm/day in cycles where ovarian stimulation is done [4]. It’s a common finding that the administration of exogenous gonadotropins causes the growth of multiple follicles. However, studies have shown that the threshold of FSH causing follicular growth may vary among different follicles, even within a single cohort [5]. This is the explanation for the inconsistent and asynchronous follicular growth observed in some cycles [5]. The incidence of poor ovarian response (POR) during ovarian stimulation has been reported to range from 5.6 to 35.1% [6]. Patients with POR typically have higher basal FSH levels, lower AMH levels, and fewer oocytes retrieved using conventional ovarian stimulation protocols. The incidence of POR may be attributed to advanced maternal age or iatrogenic reasons, such as ovarian surgery, pelvic adhesions, and obesity. Patients with POR have higher cycle cancellation rates and lower pregnancy rates per transfer with lower cumulative pregnancy rates per started cycle than are found in normal responders [6].

In our case, most of the follicles (in contrast to only one or two follicles as in asynchronous follicular growth) grew at a speedy rate. Therefore, the pattern of follicular growth is described as “synchronous” and “hyper-accelerated.” Such cases have never been reported previously, and this is the first instance of a SHAF case being reported. When a follicle grows at a very fast rate, the chances of premature luteinization or even follicular rupture can pose a significant challenge to the clinician. In our case, when follicles reach 18-20 mm in size, the stimulation day is only five, and we cannot yet initiate pituitary downregulation regimes. Given that the other cohort of follicles was tiny and only a few, we decided to proceed with the trigger.

Luteinization, by definition, requires a surge of LH, which acts on granulosa cells, causing them to increase in size and develop a vacuolated appearance with characteristic yellow pigment known as lutein. The term “premature luteinization” refers to an early LH surge that results in a rise in serum progesterone levels. In our case, the serum progesterone level of 0.6 ng/ml indicates that there was no premature LH surge, distinguishing this case from premature luteinization [7,8].

Double stimulation, also referred to as the DuoStim protocol, is especially designed for the poor responders [9]. In this protocol, just a few days after the conclusion of the first stimulation cycle, further stimulation is re-initiated irrespective of the menstrual day. The DuoStim protocol is also indicated in situations where the female is planned for chemotherapy [10]. In our case, we also explored the DuoStim protocol, achieving good results in terms of oocyte retrieval and the production of high-quality embryos.

## Conclusions

SHAF growth is a unique and potentially underappreciated phenomenon in the realm of controlled ovarian stimulation cycles. This condition is marked by a rapid and uniform ovarian response that challenges traditional stimulation protocols. If not recognized promptly, SHAF can lead to complications, including premature luteinization and early follicle rupture. Our case highlights the crucial importance of early and vigilant monitoring during ovarian stimulation cycles to minimize the risks of OHSS and ensure optimal oocyte quality. By embracing a flexible protocol approach, such as tailored gonadotropin dosing, timely adjustments of trigger injections, and real-time ultrasonographic assessments, one may salvage the stimulated cycle and hence justify the old saying “one size does not fit all.” Looking ahead, future research must prioritize large-scale epidemiological studies to accurately assess the incidence of SHAF across diverse populations and stimulation regimens. As current insights are largely anecdotal and limited, establishing reliable biomarkers, such as trajectories of serum AMH, follicular fluid metabolomics, endocrine profiles, or genetic markers, during the early phases of stimulation could pave the way for predictive modeling and proactive interventions.
